# Pediatric Moyamoya Disease and Syndrome in Italy: A Multicenter Cohort

**DOI:** 10.3389/fped.2022.892445

**Published:** 2022-05-06

**Authors:** Chiara Po', Margherita Nosadini, Marialuisa Zedde, Rosario Pascarella, Giuseppe Mirone, Domenico Cicala, Anna Rosati, Alessandra Cosi, Irene Toldo, Raffaella Colombatti, Paola Martelli, Alessandro Iodice, Patrizia Accorsi, Lucio Giordano, Salvatore Savasta, Thomas Foiadelli, Giuseppina Sanfilippo, Elvis Lafe, Federico Zappoli Thyrion, Gabriele Polonara, Serena Campa, Federico Raviglione, Barbara Scelsa, Stefania Maria Bova, Filippo Greco, Duccio Maria Cordelli, Luigi Cirillo, Francesco Toni, Valentina Baro, Francesco Causin, Anna Chiara Frigo, Agnese Suppiej, Laura Sainati, Danila Azzolina, Manuela Agostini, Elisabetta Cesaroni, Luigi De Carlo, Gabriella Di Rosa, Giacomo Esposito, Luisa Grazian, Giovanna Morini, Francesco Nicita, Francesca Felicia Operto, Dario Pruna, Paola Ragazzi, Massimo Rollo, Alberto Spalice, Pasquale Striano, Aldo Skabar, Luigi Alberto Lanterna, Andrea Carai, Carlo Efisio Marras, Renzo Manara, Stefano Sartori

**Affiliations:** ^1^Paediatric Neurology and Neurophysiology Unit, Department of Women's and Children's Health, University Hospital of Padova, Padova, Italy; ^2^Department of Women's and Children's Health, University of Padova, Padova, Italy; ^3^Unit of Pediatrics, AULSS 2 Marca Trevigiana, Ca' Foncello Hospital, Treviso, Italy; ^4^Neuroimmunology Group, Paediatric Research Institute “Città della Speranza”, Padova, Italy; ^5^Neurology Unit, Stroke Unit, Azienda Unità Sanitaria Locale-IRCCS di Reggio Emilia, Reggio Emilia, Italy; ^6^Neuroradiology Unit, Arcispedale S. Maria Nuova AUSL Reggio Emilia – IRCCS, Reggio Emilia, Italy; ^7^Pediatric Neurosurgery Unit, Department of Neuroscience, Santobono-Pausilipon Children's Hospital, Naples, Italy; ^8^Pediatric Neuroradiology, Department of Neuroscience, Santobono-Pausilipon Children's Hospital, Naples, Italy; ^9^Department of Neuroscience, Children's Hospital Anna Meyer, University of Firenze, Firenze, Italy; ^10^Clinic of Pediatric Hematology Oncology, Department of Women's and Children's Health, University of Padova, Padova, Italy; ^11^Child Neurology and Psychiatry Unit, ASST Spedali Civili of Brescia, Brescia, Italy; ^12^Pediatric Clinic, IRCCS Policlinico San Matteo Foundation, University of Pavia, Pavia, Italy; ^13^Department of Diagnostic and Interventional Radiology and Neuroradiology, IRCCS Policlinico San Matteo, Pavia, Italy; ^14^Department of Diagnostic and Interventional Radiology and Neuroradiology, IRCSS Policlinico San Matteo, Pavia, Italy; ^15^Neuroradiology - Department of Odontostomatologic and Specialized Clinical Sciences, Università Politecnica delle Marche, Ancona, Italy; ^16^Neuroradiology Unit, University Hospital “Ospedali Riuniti di Ancona, ” Università Politecnica delle Marche, Ancona, Italy; ^17^Hospital Neuropsychiatry Service, ASST Rhodense, Milan, Italy; ^18^Department of Pediatric Neurology, Vittore Buzzi Children's Hospital, Milan, Italy; ^19^Pediatric Clinic, Department of Clinical and Experimental Medicine, University Hospital A.U.O. “Policlinico-San Marco” of Catania, Catania, Italy; ^20^IRCCS Istituto delle Scienze Neurologiche di Bologna, UOC Neuropsichiatria dell'età Pediatrica, Bologna, Italy; ^21^Department of Medical and Surgical Sciences (DIMEC), University of Bologna, Bologna, Italy; ^22^Neuroradiology Unit, IRCSS “Istituto delle Scienze Neurologiche di Bologna, ” Ospedale Bellaria, Bologna, Italy; ^23^Academic Neurosurgery, Department of Neurosciences, University of Padova, Padova, Italy; ^24^Neuroradiology, Department of Neurological Sciences, University of Padova, Padova, Italy; ^25^Unit of Biostatistics, Epidemiology and Public Health, Department of Cardiac, Thoracic and Vascular Sciences, University of Padova, Padova, Italy; ^26^Pediatric Section, Department of Medical Sciences, University of Ferrara, Ferrara, Italy; ^27^Department of Pediatrics, Regina Margherita Children's Hospital, Torino, Italy; ^28^Department of Child Neuropsychiatry, University Hospital Ospedali Riuniti, Ancona, Italy; ^29^Unit of Child Neurology and Psychiatry, Department of Human Pathology of the Adult and Developmental Age, Messina, Italy; ^30^Pediatric Neurosurgery Unit, Department of Neuroscience and Neurorehabilitation, Bambino Gesù Children's Hospital, Roma, Italy; ^31^Department of Pediatrics, Institute for Maternal and Child Health - IRCCS “Burlo Garofolo”, Trieste, Italy; ^32^Unit of Neuromuscular and Neurodegenerative Disorders, Department of Neurosciences, Bambino Gesù Children's Hospital, IRCCS, Rome, Italy; ^33^Child Neurology Division, Department of Pediatrics, Sapienza University, Rome, Italy; ^34^Child and Adolescent Unit, Department of Medicine, Surgery and Dentistry, University of Salerno, Salerno, Italy; ^35^Neurology and Epileptology Unit, Department of Pediatric, ARNAS Brotzu, Cagliari, Italy; ^36^Department of Neurosurgery, “Regina Margherita” Children's Hospital, Torino, Italy; ^37^Interventional Radiology Unit, Department of Imaging, Bambino Gesù Children's Hospital (IRCCS), Rome, Italy; ^38^Department of Maternal Sciences, Sapienza University, Rome, Italy; ^39^Pediatric Neurology and Muscular Diseases Unit, IRCCS “Istituto Giannina Gaslini”, Genova, Italy; ^40^Department of Neurosciences, Rehabilitation, Ophthalmology, Genetics, Maternal and Child Health, University of Genova, Genova, Italy; ^41^Department of Neurosurgery, Ospedale Papa Giovanni XXIII, Bergamo, Italy; ^42^Neuroradiology Unit, Department of Neurological Sciences, University of Padova, Padova, Italy; ^43^Department of Neuroscience, University of Padova, Padova, Italy; ^44^Neuroimmunology Group, Paediatric Research Institute “Città della Speranza”, Padova, Italy

**Keywords:** moyamoya, transient ischemic attack, cerebrovascular events, arteriopathy, indirect revascularization, aspirin, headache, stroke

## Abstract

**Background:**

Moyamoya is a rare progressive cerebral arteriopathy, occurring as an isolated phenomenon (moyamoya disease, MMD) or associated with other conditions (moyamoya syndrome, MMS), responsible for 6–10% of all childhood strokes and transient ischemic attacks (TIAs).

**Methods:**

We conducted a retrospective multicenter study on pediatric-onset MMD/MMS in Italy in order to characterize disease presentation, course, management, neuroradiology, and outcome in a European country.

**Results:**

A total of 65 patients (34/65 women) with MMD (27/65) or MMS (38/65) were included. About 18% (12/65) of patients were asymptomatic and diagnosed incidentally during investigations performed for an underlying condition (incMMS), whereas 82% (53/65) of patients with MMD or MMS were diagnosed due to the presence of neurological symptoms (symptMMD/MMS). Of these latter, before diagnosis, 66% (43/65) of patients suffered from cerebrovascular events with or without other manifestations (ischemic stroke 42%, 27/65; TIA 32%, 21/65; and no hemorrhagic strokes), 18% (12/65) of them reported headache (in 4/12 headache was not associated with any other manifestation), and 26% (17/65) of them experienced multiple phenotypes (≥2 among: stroke/TIA/seizures/headache/others). Neuroradiology disclosed ≥1 ischemic lesion in 67% (39/58) of patients and posterior circulation involvement in 51% (30/58) of them. About 73% (47/64) of patients underwent surgery, and 69% (45/65) of them received aspirin, but after diagnosis, further stroke events occurred in 20% (12/61) of them, including operated patients (11%, 5/47). Between symptom onset and last follow-up, the overall patient/year incidence of stroke was 10.26% (IC 95% 7.58–13.88%). At last follow-up (median 4 years after diagnosis, range 0.5–15), 43% (26/61) of patients had motor deficits, 31% (19/61) of them had intellectual disability, 13% (8/61) of them had epilepsy, 11% (7/61) of them had behavioral problems, and 25% (13/52) of them had mRS > 2. The proportion of final mRS > 2 was significantly higher in patients with symptMMD/MMS than in patients with incMMS (*p* = 0.021). Onset age <4 years and stroke before diagnosis were significantly associated with increased risk of intellectual disability (*p* = 0.0010 and *p* = 0.0071, respectively) and mRS > 2 at follow-up (*p* = 0.0106 and *p* = 0.0009, respectively).

**Conclusions:**

Moyamoya is a severe condition that may affect young children and frequently cause cerebrovascular events throughout the disease course, but may also manifest with multiple and non-cerebrovascular clinical phenotypes including headache (isolated or associated with other manifestations), seizures, and movement disorder. Younger onset age and stroke before diagnosis may associate with increased risk of worse outcome (final mRS > 2).

## Introduction

Moyamoya is a progressive cerebral arteriopathy characterized by the association of two related phenomena: the development of stenosis and occlusions on the terminal portion of the internal carotid artery and/or middle and anterior cerebral arteries; and the growth of a network of collaterals in the proximity of the stenotic vessels (1–3). This condition predisposes to cerebrovascular ischemic events due to the reduced cerebral reserve, and hemorrhagic events due to the fragility of the new vessels (1, 2). Moyamoya arteriopathy can be isolated (moyamoya disease, MMD) or associated with other neurologic or extraneurologic conditions (moyamoya syndrome, MMS) (1).

Moyamoya is rare in Western countries, with estimated incidence rate lower than 0.1 person/year in Caucasians, including both adults and children (4). Despite this, moyamoya is responsible for about 20–25% of pediatric strokes due to arteriopathy (5–7), and overall is associated with 6–10% of all childhood strokes and transient ischemic attacks (TIAs), with high risk of recurrence (8–12). Due to the rarity of the disease, limited data are available on MMD and MMS in European children.

This retrospective multicenter study was conducted to characterize presentation, disease course, treatment, neuroradiology features, and outcomes of pediatric-onset MMD and MMS in Italy.

## Materials and Methods

### Clinical Phenotyping

The study was designed as an Italian retrospective multicenter survey, realized on behalf of the Italian Society of Pediatric Neurology (SINP). An ad-hoc questionnaire to collect clinical information was emailed to each SINP member. Other specialists, including members of the Italian Societies of Neurosurgery and of Neuroradiology, and pediatric hematologists of the Italian Association of Pediatric Hematology Oncology, were involved through personal contacts by the study coordinators. Data collection was from December 2015 to August 2018.

Inclusion criteria were diagnosis of MMD or MMS according to the international consensus diagnostic criteria (2, 13); and symptom onset or incidental diagnosis before age 18 years.

Clinical presentation of moyamoya was categorized according to the international consensus “disease types” (2): TIA, recurrent TIA, ischemic stroke (IS), hemorrhagic stroke, headache, epilepsy, and others. Asymptomatic patients diagnosed incidentally during investigations performed for other medical conditions were considered as a distinct group (incidental MMS, “incMMS”), with the aim of identifying possible differences from patients with MMD or MMS who had moyamoya-related symptoms at the time of diagnosis (“symptMMD,” “symptMMS,” collectively denominated “symptMMD/MMS”). Moreover, the categories of “single” and “multiple phenotypes” were introduced to differentiate patients with history of one or more than one disease type at the time of diagnosis. Outcome was investigated with open fields about “cognitive,” “motor,” “memory,” and “behavior” problems and by using the modified Rankin Scale (mRS), a scale initially created for stroke but now widely used in pediatric neurology, despite its limitations in detecting non-motor issues (14). We operatively defined poor outcome as mRS > 2.

Available neuroimaging scans were reviewed by an experienced pediatric neuroradiologist in order to evaluate unilateral vs. bilateral involvement, anterior vs. posterior circulation distribution, presence of ischemic or hemorrhagic lesions, and micro-bleedings.

The study complied with the general ethical requirements for retrospective observational studies. In particular, no experimental interventions were performed, and patient identity cannot be retrieved from the manuscript.

### Statistic Analysis

Descriptive statistic was used to organize and present data.

When comparing the three groups of patients (symptMMS, symptMMD, and incMMS) with respect to quantitative variables, the Mann–Whitney test followed by the Dwass–Steel–Critchlow–Fligner method for multiple pairwise comparisons were used. For categorical variables, the groups were compared with Fisher's exact test followed by pairwise comparisons correcting the *p*-values with Bonferroni's method.

Incidence of events in the follow-up was estimated along with 95% confidence interval, with a Poisson model considering the natural logarithm of follow-up time as offset parameter. The statistical analysis was performed with SAS 9.4 (SAS Institute Inc., Cary, NC, USA) for Windows.

## Results

### Demographics and Disease Presentation

Sixty-five patients were included (34/65, 52% women). Mean age at moyamoya diagnosis was 7.4 years (median 7, range 0.4–24.5). A total of 27/65 (42%) patients had MMD and 38/65 (58%) patients had MMS. Among patients with MMS, 22/38 (58%) patients had underlying genetic conditions (most frequent being neurofibromatosis type 1 in 6/22, sickle cell disease in 5/22, and other suspected genetic multisystemic syndromes in 8/22; Table 1A).

**Table 1:** Descriptive demographics, MMD/MMS data (1A) clinical and outcome data (1B) in the total cohort of pediatric-onset moyamoya patients (*n* = 65).

**Total cohort of patients with pediatric-onset MMD or MMS (*****n*** **=** **65)**					
**Table 1A: Demographics and MMD/MMS data**					
**Demographics**					
Referring centers		18 (Northern Italy: 7/18; Central Italy: 7/18; Southern Italy: 4/18; mean 3 patients/center, range 1–14)			
Females		34/65 (52%)			
Mean age at diagnosis		7.4 years (median 7, range 0.4–24.5; d.a. 65/65)			
Race		Caucasian 51/65 (78%), Asian 9/65 (14%), African 5/65 (8%)			
Familial cases		4/65 (6%)			
**MMD/MMS**					
Moyamoya disease (MMD)		27/65 (42%)			
Moyamoya syndrome (MMS)		38/65 (58%)	Underlying conditions in patients with MMS^*^(***n*** **=** **38)**		
			**Genetic conditions** ***n*** **=** **22**: Neurofibromatosis type 1 6/22 (molecularly proven 1/6), sickle cell disease 5/22 (molecularly proven 4/5; unknown 1/5), Marfan disease 2/22 (molecularly proven 0/2), Down syndrome 1/22 (molecularly proven 1/1), suspected genetic multisystemic syndrome 8/22 (various associations of dysmorphic features and cutaneous, ocular, endocrine or neurodevelopmental defects)		
			**Infectious diseases** ***n*** **=** **3**: congenital CMV infection 1/3, post-natal CMV infection 1/3, cerebral tuberculosis 1/3		
			**Renal or renovascular diseases** ***n*** **=** **4**: renal arterial stenosis 3/4, renal cysts 1/4		
			**Neurologic or neurodevelopmental disorders** ***n*** **=** **6**: pre-existing developmental delay of unknown origin 3/6, epilepsy 2/6, neurosensorial hearing loss 1/6		
			**Heart diseases** ***n*** **=** **8**: atrial septal defects and/or persistent ductus arteriosus 3/8, complete atrioventricular septal defect 1/8, coarctation of the aorta 1/8, heart valve diseases 3/8		
			**Endocrine diseases** ***n*** **=** **7**: GH deficit 4/7, panhypopituitarism 1/7, hypothyroidism 1/7, precocious puberty 1/7		
			**Post-actinic** ***n*** **=** **2**: craniopharyngioma 1/2, optic chiasm ganglioglioma 1/2		
**Table 1B: Clinical data at diagnosis and Outcome at last follow-up**					
**Clinical data at diagnosis**					
incMMS (asymptomatic, incidentally diagnosed)		12/65 (18%)			
symptMMD/MMS (neurological symptoms before diagnosis)		53/65 (82%)	**Clinical data at diagnosis in symptMMD/MMS patients (*****n*** **=** **53)**		
			Mean age at symptom onset 5.4 years (median 4, range 0.4–16; d.a. 53/53)		
			Mean delay from symptom onset to moyamoya diagnosis 1.4 years (median 7 days, range 0–20 years; d.a. 53/53)		
			**First-ever clinical manifestation**		
			Ischemic stroke 19/65 (29%)		
			TIA 17/65 (26%)		
			Seizures 9/65 (14%)		
			Headache 8/65 (12%)		
			**Single phenotype at the time of diagnosis**		36/65 (55%)
			Isolated ischemic stroke 17/65 (26%)		
			Isolated TIA/TIAs 12/65 (18%)		
			Isolated headache 4/65 (6%)		
			Isolated seizures 3/65 (5%)		
			**Multiple phenotypes at the time of diagnosis** 17/65 (26%)		
			Headache and cerebrovascular events (TIA/stroke) +/– other 6/65 (9%)		
			Seizures and cerebrovascular events (TIA/stroke) 5/65 (8%)		
			TIA and stroke 3/65 (5%)		
			Headache, seizures and stroke OR seizures and headache OR seizures and other 3/65 (5%)		
**Outcome at last follow-up**					
Mean duration of follow-up after diagnosis		5.1 years (median 4, range 0.5–15; d.a. 61/65)			
	Final mRS 0 20/52 (39%)				
	Final mRS 1 12/52 (23%)				
	Final mRS 2 7/52 (13%)				
	Final mRS 3 9/52 (17%)				
	Final mRS 4–5 4/52 (8%)				
Motor deficits at last follow-up		26/61 (43%)			
Intellectual disability at last follow-up		19/61 (31%)			
Epilepsy at last follow-up		8/61 (13%)			
Memory disturbances at last follow-up		7/61 (11%)			
Behavioral problems at last follow-up		7/61 (11%) (anxiety disorder 1/7, aggressive behavior 2/7, attention deficit and hyperactivity 3/7, sleep disturbance 2/7)			
Movement disorder at last follow-up		0/61 (0%)			

*Detailed data are provided for the subgroup of MMS (n = 38) and for the subgroup of patients with MMD or MMS diagnosed due to the presence of neurological symptoms before diagnosis (symptMMD/MMS, n = 53)*.

*CMV, cytomegalovirus; d.a., data available; GH, growth hormone; MMD, moyamoya disease; MMS, moyamoya syndrome; mRS, modified Rankin Scale; incMMS, patients with MMS asymptomatic at the time of diagnosis, diagnosed incidentally during investigations carried out for the underlying condition; symptMMD/MMS, patients with MMD or MMD diagnosed due to the presence of neurological symptoms; TIA, transient ischemic attack*.

*∧Some patients had more than one of the listed underlying conditions*.

*Genetic tests were carried out in 17/38 patients with MMS (no genetic tests done in 17/38, data not available in 4/38), and resulted negative in 7 patients (MELAS n = 1; Axenfeld Rieger n = 1; FISH for NF1 n = 1; karyotype; n = 1 arrayCGH and karyotype n = 1; arrayCGH n = 2), and positive or with uncertain significance in the remaining 10 patients (arrayCGH del 4q24 and del 9p24.1 n = 1; arrayCGH 15q13.2q13.3 n = 1; arrayCGH del 16p13.2p1 of maternal origin, del 6p25.3 + dup 6p24.3-p25.3 de novo n = 1; NF1 nonsense W221X mutation of exon 5 n = 1; SCD mutation n = 4; coagulation factors mutations n = 2).

*Among 27 patients with MMD, only 3/27 patients underwent genetic tests (21/27 patients did not undergo any genetic test, whereas in 3/27 patients, these data were not available), resulted negative (arrayCGH n = 3)*.

**Figure 1:**
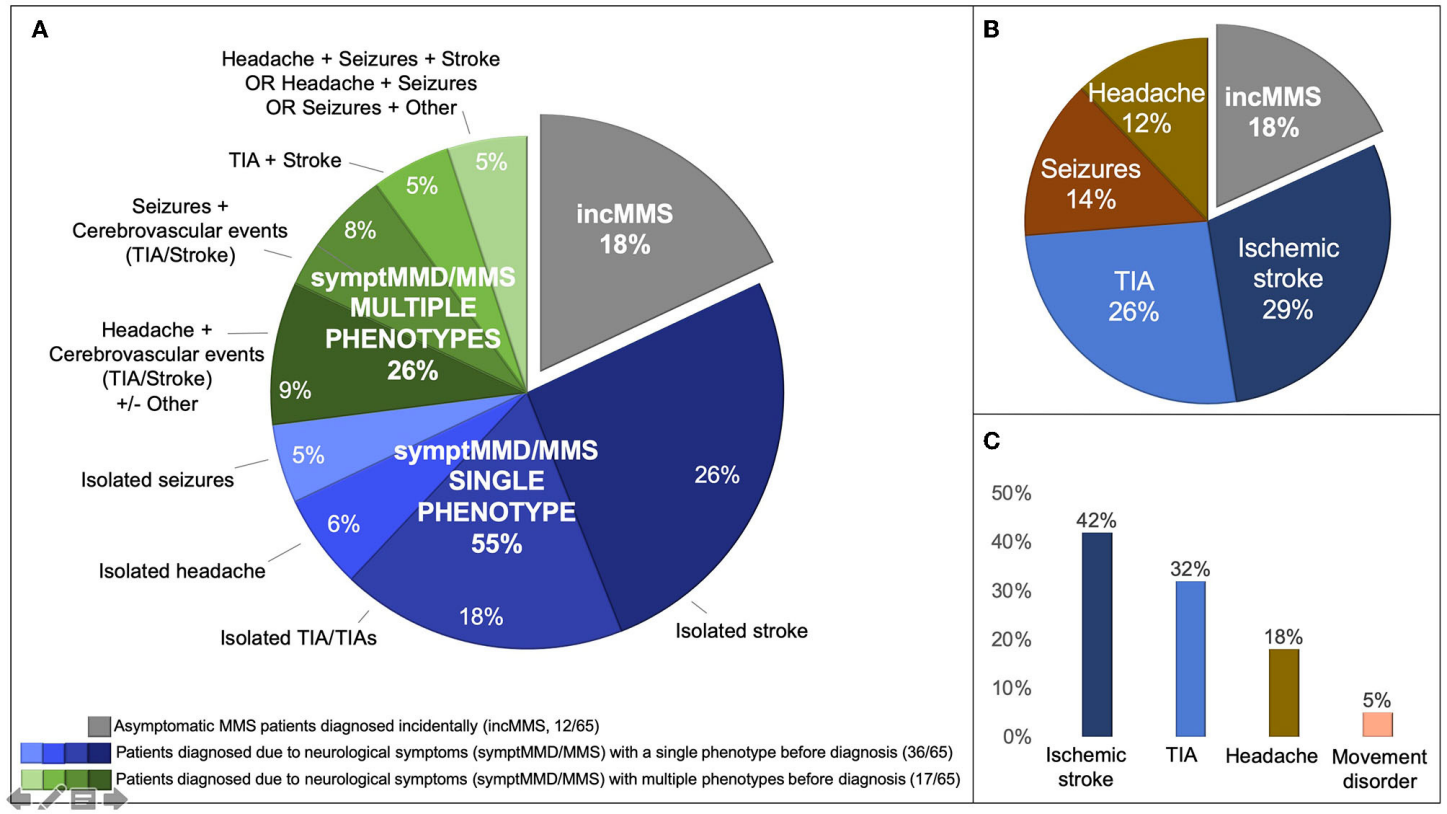
Clinical data. **(A)** Clinical phenotype in the total pediatric MMD and MMS cohort at the time of diagnosis (*n* = 65): incidentally diagnosed MMS in gray, asymptomatic at the time of diagnosis (incMMS) (*n* = 12) and symptomatic patients with MMD or MMS (symptMMD/MMS, *n* = 53) in blue shades (single phenotype at the time of diagnosis, *n* = 36) and green shades (multiple phenotypes at the time of diagnosis, *n* = 17). Single or multiple phenotypes at the time of diagnosis was categorized according to the presence of one or more than one international consensus “disease types” (2): transient ischemic attack (TIA), recurrent TIA, ischemic stroke, hemorrhagic stroke, headache, epilepsy, and others (see Section “Methods”). **(B)** First-ever clinical manifestation in 53 patients with MMD or MMS with moyamoya-related symptoms at diagnosis (symptMMD/MMS). Ischemic stroke and TIA were the most frequent clinical manifestations at symptom onset. **(C)** Frequency of previous or active neurological manifestations in 53 patients with MMD or MMS with moyamoya-related symptoms at diagnosis (symptMMD/MMS). In patients with symptMMD/MMS, at the time of diagnosis 81% had experienced previous or recent cerebrovascular events (TIA, ischemic stroke).

Twelve of the 65 patients (18%), all patients with MMS, were diagnosed incidentally during investigations performed for an underlying medical conditions (incMMS), whereas the remaining 53 patients (82%) with MMD or MMS were diagnosed due to the presence of neurological symptoms (symptMMD/MMS; Table 1A and Figure 1A).

Age at diagnosis was significantly lower in the 53 symptomatic patients (symptMMD/MMS) than in the 12 patients diagnosed incidentally (incMMS) (*p* = 0.0467) (Table 2).

**Table 2:** Comparison of disease presentation, course, and outcomes in children with symptomatic moyamoya disease (symptMMD), symptomatic moyamoya syndrome (symptMMS), and asymptomatic incidentally diagnosed moyamoya syndrome (incMMS).

	**Total cohort** **(*n =* 65)**	**symptMMD** **(*n =* 27)**	**symptMMS** **(*n =* 26)**	**incMMS** **(*n =* 12)**	***p-*value**
**Age at symptom onset (years)** **Mean (SD)** **Median (range)**	5.4 (4.1) 4 (0.4-16) (*n =* 53)	4.9 (3.4) 4.8 (0.4-13) (*n =* 27)	5.8 (4.7) 3.2 (1.2-16) (*n =* 26)	nd	0.68^a^
**Age at diagnosis (years)** **Mean (SD)** **Median (range)**	7.4 (5.2) 7 (0.4-24.6) (*n =* 65)	6.7 (4.4) 7 (0.4-19.6) (*n =* 27)	6.9 (6) 4.9 (1.3-24.6) (*n =* 26)	10.2 (4.2) 10.7 (3-17.8) (*n =* 12)	**0.0467^b^**
Ischemic stroke before diagnosis^*^	27/53 (50.9%)	13/27 (48.1%)	14/26 (53.8%)	nd	0.78^c^
**Ischemic stroke after diagnosis**	12/65 (18.5%)	4/27 (14.8%)	8/26 (30.8%)	0/12 (0.0%)	0.056^c^
**Neurosurgery**	47/64 (73.4%)	20/27 (74.1%)	20/25 (80.0%)	7/12 (58.3%)	0.40^c^
**Acetylsalicylic acid (ASA) therapy**	45/60 (75.0%)	20/26 (76.9%)	21/25 (84.0%)	4/9 (44.4%)	0.070^c^
**Intellectual disability at last follow-up**	19/59 (32.2%)	7/23 (30.4%)	11/25 (44.0%)	1/11 (9.1%)	0.11^c^
**mRS** **>2** **At last follow-up**	14/52 (26.9%)	4/21 (19.0%)	10/22 (45.4%)	0/9 (0.0%)	**0.021** ^c^

*In the 53 patients diagnosed due to the presence of neurological symptoms (symptMMD and symptMMS), age at diagnosis was significantly lower and proportion of mRS > 2 at last follow-up significantly higher compared to the 12 asymptomatic patients diagnosed incidentally (incMMS)*.

*symptMMD, moyamoya disease (with moyamoya-related symptoms at the time of diagnosis); symptMMS, moyamoya syndrome (with moyamoya-related symptoms at the time of diagnosis); incMMS, incidentally diagnosed MMS (patients diagnosed with moyamoya incidentally during investigations carried out for an underlying condition, in the absence of moyamoya-related symptoms at diagnosis); mRS, modified Rankin Scale*.

* *In 26/27 patients with ischemic stroke at diagnosis, stroke was the neurological event that lead to medical attention and to the diagnosis; only in 1/27 cases stroke occurred previously, and the diagnosis was made later due to the onset of other neurological symptoms*.

a *Mann–Whitney test*.

b *Kruskal–Wallis test*.

c *Fisher's exact test. The bold text in the p-value column indicates statistically significant values*.

The first-ever clinical manifestation was IS in 19/65 (29%), TIA in 17/65 (26%), seizures in 9/65 (14%), and headache in 8/65 (12%) patients (Table 1A and Figure 1B; all symptMMD/MMS).

At the time of diagnosis, 36/65 (55%) patients had a history of a single clinical phenotype, whereas 17/65 (26%) patients had a history of multiple phenotypes (all symptMMD/MMS) (Table 1B and Figure 1A), but none of the patients had hemorrhagic stroke.

Overall, by the time of diagnosis, cerebrovascular events (TIA and IS), whether isolated or associated with other symptoms, occurred or had occurred in 43/65 patients (66%) (all symptMMD/MMS) (Figure 1C): TIA/TIAs in 21/65 (32%) patients (estimated mean age at TIA presentation: 6 years), and IS in 27/65 (42%) patients (mean age at stroke presentation: 4.1 years; median 2.75, range 0.4–14). Among patients with symptMMD/MMS, stroke before diagnosis was more frequent among patients with symptom onset before age 24 months (7/10, 70%) than in those with later onset (20/43, 47%) (Table 3 and Supplementary Material S1).

**Table 3:** Comparison between the subpopulations of symptomatic children with MMD or MMS (symptMMD/MMS) with symptoms onset respectively before and after 24 months of age.

	**Symptoms onset before 24 months of age**	**Symptoms onset after 24 months of age**
**Number of patients**	10/53 (19%)	43/53 (81%)
**M:F**	7:3	20:23
**MMD:MMS**	6:4	21:22
**Mean age at symptoms onset**	1.2 years (median 1.3)	6.3 years (median 5.2)
**Mean age at diagnosis**	1.5 years (median 1.4)	7.7 years (median 7)
**Ischemic stroke before diagnosis**	7/10 (70%)	20/43 (47%)
**Posterior circulation involvement at neuroimaging**	7/9 (78%)	22/37 (59%)
**Neurosurgery**	10/10 (100%)	30/42 (71%)
**Acetylsalicylic acid (ASA)**	8/10 (80%)	33/41 (80%)
**Cerebrovascular events after diagnosis (TIA/stroke)**	7/10 (70%)	17/39 (44%)
**Intellectual disability at follow-up**	5/9 (56%)	13/49 (27%)
**mRS at follow-up (mean)**	1.87	1.44
**mRS** **>2 at follow-up**	3/8 (37%)	10/35 (29%)

*In patients with symptom onset before 24 months of age, the frequency of stroke before diagnosis, of cerebrovascular events after diagnosis and the proportion of patients with intellectual disability at last follow-up were higher than in patients with a later onset*.

*F, females; M, males; MMD, moyamoya disease; MMS, moyamoya syndrome; mRS, modified Rankin Scale; TIA, transient ischemic attack*.

The presence or history of headache before diagnosis was reported in 12/65 (18%) patients (tension-type headache in 1/12 patients, and the remaining patients had migraine associated with tension-type headache) (all symptMMD/MMS). Of these 12 patients, 4/12 patients had isolated headache (mean 32 months between headache onset and moyamoya diagnosis; median 24, range 0–72), 7/12 patients had headache followed by other clinical manifestations (mean 42 months between headache onset and moyamoya diagnosis; median 16, range 1–120), and 1/12 patients had headache occurring after an undiagnosed IS (25 years between stroke and moyamoya diagnosis).

Movement disorder (dyskinetic, choreiform, and dystonic pattern) was reported in 3/65 (5%) patients before diagnosis (age range 1.25–13.3 years) (all symptMMD/MMS), associated with different clinical phenotypes (mainly IS).

### Surgical Treatment

47/64 (73%) patients underwent neurosurgery, between 1995 and 2017: 42/47 (89%) indirect revascularization, 4/47 (9%) combined techniques, and 1/47 (2%) direct revascularization.

Mean age at surgery was 6.1 years (median 4.8, range 0.4–17.7) and at mean 15.7 months after diagnosis (median 3, range few days to 8 years; data available in 39/47 patients). Surgery was carried out within 1 month after diagnosis in 10/47 operated patients (21%).

Overall, stroke at diagnosis had been reported in 23/47 operated patients (49%), and in 7/10 (70%) of the patients operated within 1 month after diagnosis (mean age at diagnosis in this latter subgroup: 3.5 years).

Surgery complications occurred in 3/47 patients (6%): one patient had a severe intra-operatory ischemic damage; one patient had a mild frontal blood suffusion; and one patient had subdural hematoma.

A second intervention was carried out in 22/47 patients (47%) (21/22 patients to complete the first surgery, and 1/22 patients to evacuate a subdural hematoma). Complications of the second surgery occurred in 4/22 (18%) patients: removal of a iatrogenic foreign body in one patient; liquor fistula requiring lumbar puncture in one patient; cerebral edema due to revascularization in one patient; and asymptomatic parenchymal hematoma with spontaneous resolution in one patient.

### Medical Treatment

Acetylsalicylic acid (ASA) was used in 45/65 patients (69%) overall, including 38/47 (80.9%) operated patients. Transient adverse events occurred in 3/45 patients overall (petechiae, cutaneous reactions). ASA was started within 1 month after diagnosis in 26/45 (58%) patients, between 4 and 12 months in 4/45 (9%) patients, and more than 1 year later in 5/45 (11%; data available for 35/45 patients).

Unfractioned/low-molecular-weight heparin (LMWH) was used in 5/65 (8%) patients (4 patients were rapidly shifted to ASA; and 1 patient continued LMWH for 6 months).

Flunarizine was used in 6/65 patients (9%) (adverse events in 1/6 patients: weight gain), and calcium blockers were used in 3/65 (5%) patients (no adverse events).

Antiseizure medications were used in 30/65 patients (46%).

Among the 5 patients with sickle cell disease, 3 patients received hematopoietic stem cell transplantation, and 1 patient started red cell exchange transfusion after an IS.

### Neuroradiology

At least one neuroimaging scan was available for 58/65 patients (89%). In 45/58 patients with available information on neuroimaging timing with respect to diagnosis, 19/45 patients were with an available scan at diagnosis; and the remaining patients were performed later in the follow-up. Moyamoya diagnosis received confirmation in 58/58 patients (100%) of neuroimaging after revision by our senior pediatric neuroradiologist. Bilateral steno-occlusions and networks were observed in 47/58 patients (81%). Among patients with MMD with available neuroimaging scans, 22/24 patients (92%) had bilateral steno-occlusions and networks; and 2/24 patients (8%) had bilateral steno-occlusions and left unilateral network. Among patients with MMS with available neuroimaging scans, 25/34 patients (74%) had bilateral steno-occlusions and networks, 8/34 patients (23%) had unilateral steno-occlusion and network (2 left-sided; 6 right-sided), and 1/34 patients (3%) had bilateral steno-occlusions and left unilateral network.

**Figure 2:**
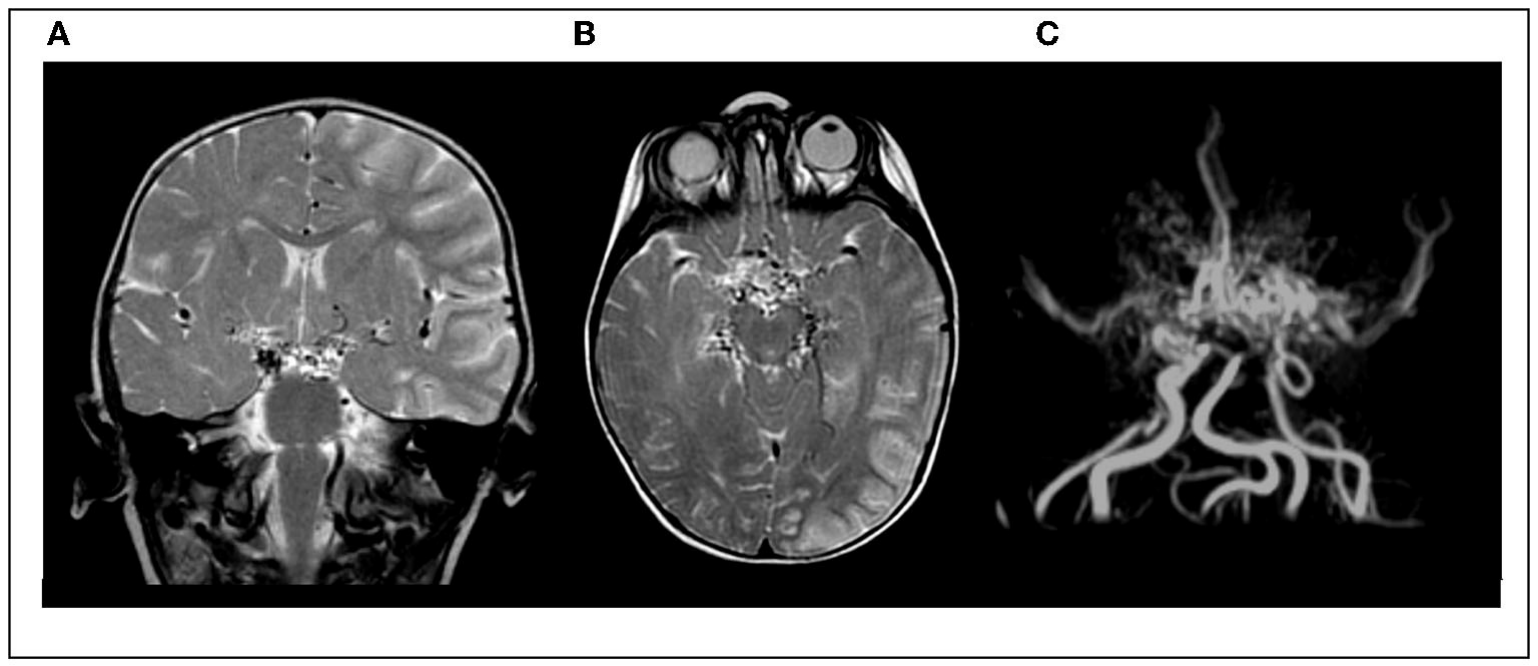
Cerebral MRI and MRA study in an 11-month-old girl with early-onset MMD, presenting with acute ischemic stroke. **(A,B)** Coronal and axial T2-w image showing left-sided cortical and subcortical infarct; multiple bilateral “flow voids” in the basal ganglia. **(C)** Magnetic resonance angiography showing typical “puff of smoke”.

Posterior circulation involvement was documented in 30/58 patients (51%), and at least one ischemic lesion in 39/58 patients (67%). Neuroimaging data are reported in Figure 2 and Supplementary Material S2.

### Disease Course After Diagnosis

Data on disease course were available for 61/65 patients.

Overall, 24/61 patients experienced cerebrovascular events (TIA and IS) after diagnosis (39%): 12/61 patients (20%) had IS (3 of them had their first stroke after diagnosis), and 12/61 patients (20%) had TIAs. The mean age at disease onset in children who experienced IS after diagnosis was lower than in children with no stroke after diagnosis (3 years vs. 6 years).

Only 1 child experienced TIA in the follow-up without having had any cerebrovascular event before diagnosis.

Among 47 patients who underwent surgery, 11/47 patients suffered from cerebrovascular events after surgery (23%): 5/47 patients had IS (11%) (5/5 patients already had stroke at the time of diagnosis), and 6/47 patients had TIAs (13%).

Based on these data, it was possible to calculate, during the entire period from symptom onset to the last follow-up, a patient/year incidence of 10.26% (IC 95% 7.58–13.88%) for stroke, 9.28% (IC 95%: 6.75–12.75%) for TIA, and a combined incidence of 19.54% (IC 15.69–24.32%) for cerebrovascular events (TIA or IS).

The involvement of posterior circulation at neuroimaging increased the incidence patients/year to 15.51% (10.78–22.32%), 9.63% (6.07–15.28%), and 25.14% (18.89–33.45%), respectively, for stroke, TIA, and cerebrovascular events.

The occurrence of cerebrovascular events after diagnosis was more frequent among patients with symptom onset before 24 months of age (7/10, 70%) than in those with symptom onset after 24 months of age (17/39, 44%) (Table 3 and Supplementary Material S1).

Headache after diagnosis was reported in 25/61 patients (41%) (migraine in 10/25 patients, tension headache in 3/25 patients, more than one headache type in 2/25 patients, and not specified in the others). Headache frequency was not uniformly reported; generally, for most patients, intensity and frequency of episodes reduced in time.

Seizures occurred in 9/61 patients after diagnosis (15%) (6/9 patients had new onset of seizures after diagnosis; 4/9 patients experienced seizure after surgery).

Movement disorders were reported in 5/61 patients after diagnosis (8%; in 1/5 patients, the symptom had started before diagnosis) (dyskinetic movements in 2/5 patients, dystonic movements in 2/5 patients, and myoclonic movements in 1/5 patients).

### Outcome

**Figure 3:**
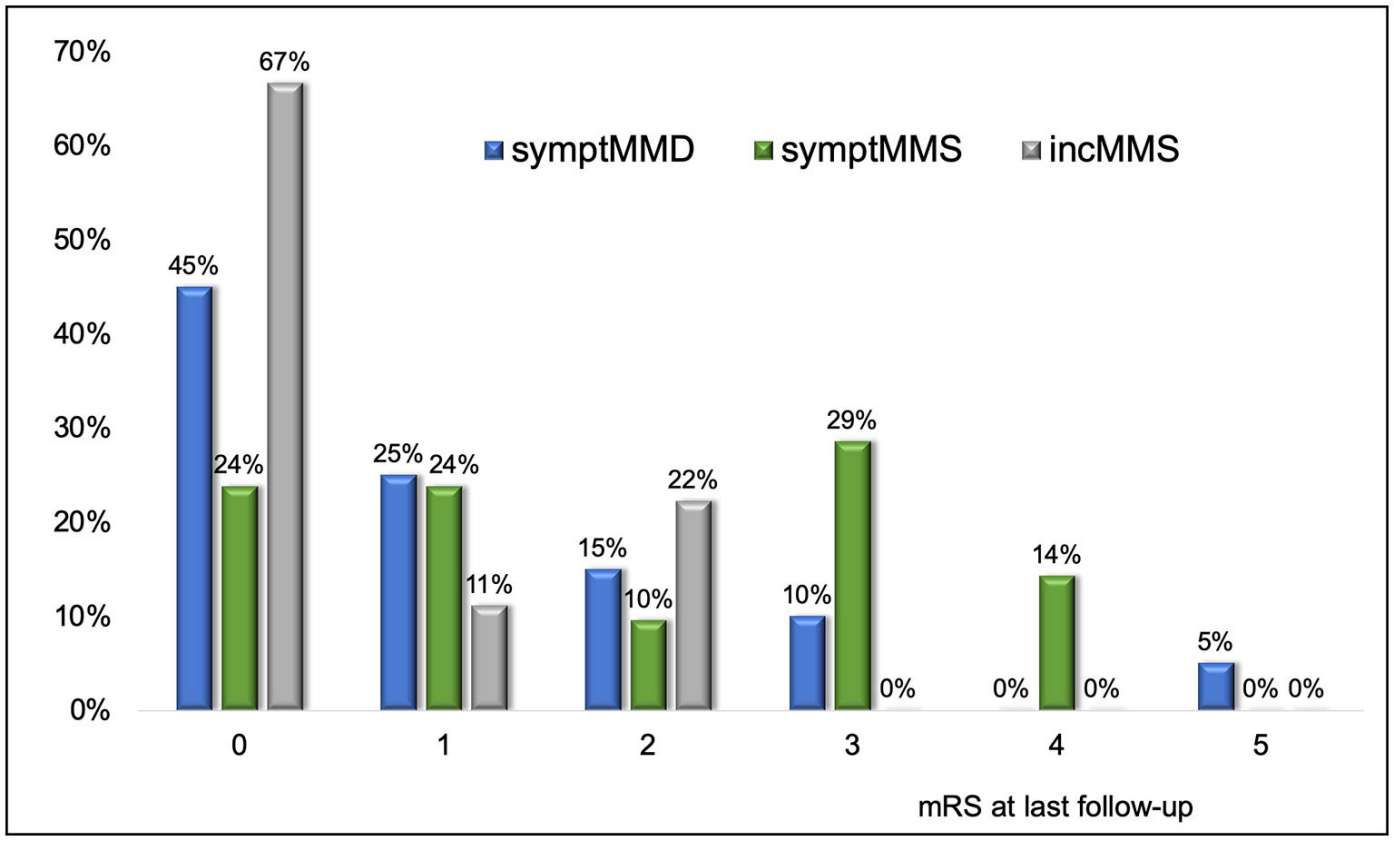
mRS at last follow-up in the total cohort of symptomatic MMD (symptMMD) (*n* = 27), symptomatic MMS (symptMMS) (*n* = 26), and asymptomatic incidentally diagnosed MMS (incMMS) (*n* = 12). The proportion of patients with mRS > 2 at last follow-up was significantly higher in symptomatic patients with MMD or MMS (symptMMD/MMS) than in asymptomatic patients diagnosed incidentally (incMMS) (*p* = 0.021) (see also Table 2).

At last follow-up, 20/52 patients (39%) had mRS 0, 10/52 patients (37%) had mRS 1–2, and 13/52 patients (25%) had mRS > 2 (Table 1B and Figure 3); no deaths occurred.

The proportion of patients with mRS > 2 at last follow-up was significantly higher in symptomatic patients (symptMMD/MMS) than in patients diagnosed incidentally (incMMS) (*p* = 0.021) (Table 2).

Age <4 years at symptom onset and IS (previous or recent) at diagnosis were both significantly associated with increased risk of intellectual disability (*p* = 0.0010 and *p* = 0.0071, respectively) and of mRS > 2 at last follow-up (*p* = 0.0106 and *p* = 0.0009, respectively) (Table 4).

**Table 4:** Predictors of main outcomes in the total study population.

	**Cerebrovascular events after diagnosis**		**Intellectual disability at last follow-up**		**mRS** **>** **2 at last follow-up**	
	*p*-value	OR (95% CI)	*p*-value	OR (95% CI)	*p*-value	OR (95% CI)
**Age** ** <4 years at symptom onset**	0.0746	2.857 (0.901–9.060)	**0.0010**	11.667 (2.696–50.490)	**0.0106**	6.966 (1.572–30.863)
**>6 months from onset to diagnosis**	0.0456	0.188 (0.036–0.968)	0.6653	0.716 (0.157–3.254)	0.0795	0.142 (0.016–1.259)
**Multiple phenotypes before diagnosis**	0.5534	1.436 (0.434–4.750)	0.4523	1.643 (0.450–5.996)	0.8439	0.857 (0.185–3.974)
**Ischemic stroke before diagnosis**	0.2232	1.925 (0.671–5.522)	**0.0071**	5.056 (1.553–16.457)	**0.0009**	16.800 (3.188–88.529)
**Neurosurgery**	0.3455	1.842 (0.518–6.550)	0.9238	1.062 (0.309–3.651)	0.4765	1.692 (0.398–7.202)
**Acetylsalicylic acid (ASA)**	0.2146	2.429 (0.598–9.862)	0.3511	1.975 (0.472–8.260)	0.1350	5.200 (0.598–45.185)
**Posterior circulation involvement**	0.1554	2.432 (0.714–8.284)	0.0919	2.933 (0.839–10.251)	0.1058	3.115 (0.786–12.346)

*Age <4 years at onset and ischemic stroke at diagnosis were both significantly associated with increased risk of intellectual disability and of final mRS > 2 at last follow-up*.

*mRS, modified Rankin Scale. The bold text in the p-value column indicates statistically significant values*.

## Discussion

We present data from the largest Italian multicenter pediatric cohort so far, second in size only to the recently reported UK cohort in the European region (15), with the aim of improving knowledge about childhood-onset moyamoya in Europe. Our cohort adds clinical knowledge on presentation, course, and outcome of this rare condition in pediatric age in a Western country, where MMD prevalence is estimated to be about 1/10 compared with Asian countries, and MMD/MMS rate is expected to be lower (0.7:1 in our cohort) than Asian countries (10:1) (2, 4).

To date, a limited number of papers have investigated moyamoya in European children, and recent published pediatric cohorts had heterogeneous populations and purposes (Supplementary Material S3), limiting their comparability (4, 15–26).

### Disease Onset and Time to Diagnosis

Symptom onset in pre-school age was frequent in our cohort, similar to other Western European and extra-European cohorts (15, 27–29) without significant differences between MMS and MMD (Table 2).

Although our study confirms some degree of diagnostic delay as in the literature (18, 29–31), this is fairly reduced compared to the previous comparable Italian report published 25 years ago (16.8 vs. 25 months) (32) (Supplementary Material S4), possibly due to higher levels of suspicion and greater availability of noninvasive cerebrovascular studies.

### Disease Presentation: Cerebrovascular Events and Beyond, a Heterogeneous Disease

Cerebrovascular events (IS and/or TIA) were by far the most frequent clinical manifestation of moyamoya in our cohort (Figure 1): 66% (43/65) of our patients experienced at least one cerebrovascular event (IS in 42%, 27/65) before diagnosis, consistent with other East Asian and Western studies (8, 15, 28, 29), while there were no hemorrhagic strokes.

The mean age at stroke presentation in our cohort was about 4 years, with a rate of stroke presentation before 4 years old of about 60%. The age at presentation of stroke due to any cause is generally older, ranging from 5.6 years old in the Swiss registry (33) to about 7–8 years in the German and the International Pediatric Stroke Study (34, 35); moreover, in the Canadian Pediatric Stroke Registry, only 33% of patients were <4 years old at diagnosis (6). These data suggest that probably moyamoya could cause IS earlier than other stroke etiologies.

In our study, about 1 out of 5 patients experienced TIA in the weeks before stroke, without receiving a formal diagnosis of moyamoya. The occurrence of one or multiple TIAs in the months prior to stroke has been previously described (29, 36). Furthermore, as already reported in another pediatric MMD cohort, in our study the age at TIA onset, about 6 years old, was lower, compared to usual onset of TIA in pediatrics (37). This highlights the importance that every child with a TIA—especially in early school age or before—receive prompt and extensive studies to rule out cerebral angiopathies.

Previous data have shown that cerebrovascular events are not the only manifestation of pediatric moyamoya, with headache at presentation, isolated, or associated with other clinical manifestations, ranging between 6.3% (8), 22% (38), and up to about 50% (29). Similarly, in our cohort, headache (isolated or with other symptoms) was reported in 18% of patients (12/65) before diagnosis and in 41% of patients (25/61) during the disease course, highlighting the importance of not overlooking headache as a neurological symptom, especially when associated with other phenomena. We could not identify a specific moyamoya headache pattern, differently to other secondary headaches (39), although the most represented was migraine-like.

Movement disorder was experienced by a subgroup of our patients, at presentation or during the disease course, but in none at last follow-up: disappearance of movement disorder during the clinical history has been previously described, even if the reasons are unclear (40). A relationship between movement disorder disappearance and surgery is likely also in our patients, independently from the mechanism (16, 40).

Moyamoya is a heterogeneous disease, with possible occurrence of multiple symptoms and disease types by the time of diagnosis (8). In our study, 26% of patients had “multiple phenotypes” at the time of diagnosis, defined as the presence of more than one disease type (TIA/TIAs, IS, hemorrhagic stroke, headache, epilepsy, and others) (2), emphasizing the importance of suspecting moyamoya in children presenting with multiple neurological manifestations.

Interestingly, a higher number of children in our cohort, compared with previous series, received diagnosis of moyamoya in the context of a genetic condition, such as sickle cell disease or neurofibromatosis type 1, despite being asymptomatic. This is possibly due to the changing epidemiology and the increased adherence to disease-specific guidelines. In particular, national guidelines for pediatric sickle cell disease recommend screening transcranial doppler from age 2 and magnetic resonance imaging from age 6 years. They also encourage considering hematopoietic stem cell transplantation in case of cerebral vasculopathy (41, 42).

### Surgical and Pharmacological Management

Surgery was extensively used in our patients, with indirect revascularization being the preferred technique according to patients' age in our cohort, and similar adverse events rate at first intervention to other international data (43–45). Interestingly, the frequency of surgery significantly increased in our cohort (surgical interventions performed from 1995 to 2017) compared with a previous Italian cohort published in 1997 (Supplementary Material S4) (32).

Regarding pharmacological management, a recent study demonstrated that antiplatelet therapy does not seem to prevent recurrent cerebral infarction, which recognizes a hemodynamic (vs. embolic) pathogenesis (46).

Nevertheless, according to different treatment recommendations for pediatric stroke, aspirin is suggested as initial therapy for children with acute ischemic stroke secondary to moyamoya, over no treatment (Grade 2C) (11) and may be considered in individuals with moyamoya after revascularization surgery or in asymptomatic individuals for whom surgery is not anticipated (Class IIb, Level of Evidence C) (10), and overall it is widely used as a long-term treatment in pediatric asymptomatic or symptomatic ischemic type MMD or MMS, especially by non-Asian physicians (47, 48). Accordingly, aspirin use was widespread in our cohort, exception made for asymptomatic patients (incMMS) (Table 2). Although this latter subgroup had a favorable course even with less frequent aspirin use, data are too limited to draw definite conclusions.

### Disease Course After Diagnosis: Persistent Stroke Risk and Disability

Both MMD and MMS are significantly associated with risk of poor neurological outcome (8, 45, 49, 50), for several reasons. First, it is striking that the risk of stroke remains also after diagnosis: in our cohort, 20% of patients experienced one or more strokes after diagnosis, including not only patients with stroke recurrence (33% of patients who suffered a first stroke before diagnosis) but also patients who presented their first stroke after diagnosis (5% of the entire study population). Arteriopathy is a known risk factor for stroke recurrence (6, 7, 35), and moyamoya vasculopathy could predispose to cerebrovascular events also in patients who presented at diagnosis with a different “disease type”.

Surgical treatment did not completely abate the risk of stroke during the disease course: among our subgroup of surgically treated patients, 11% of patients had stroke after surgery. This figure is similar to other published data (7–13%) (15, 42), but higher than other pediatric cohorts (0–3%) (22, 51, 52) (possible heterogeneity in study design and populations).

Moyamoya is also associated with variable degrees of intellectual, motor, and functional impairment at follow-up (53). The proportion of children with mRS > 2 (conventionally considered an estimate of needed support in daily life) is about 10–35% in the literature (22, 26, 36, 51, 52). In the recent UK cohort (15), 37.6% of children had mRS > 2, and 52% of them had special needs at final follow-up. Similarly, 25% of our patients had mRS > 2, and 31% of them had intellectual impairment. Early-onset disease was the factor with the most stringent significant relationship with poor outcome, as previously reported (43). As expected, also the occurrence of stroke before diagnosis was significantly correlated with cognitive and motor outcome, but the actual contribution of stroke could not be separated from the effect of age: children with early-onset disease had higher probability of suffering from stroke before the diagnosis in our cohort. A larger sample might allow separating the effect of these two variables (age at disease onset and stroke before diagnosis). Nevertheless, it is also likely that genetic factors, still partially unknown, could determine both an early-onset and a more severe disease (54, 55).

Posterior circulation involvement, although not statistically associated with outcomes, seemed to be associated with a higher rate of cerebrovascular events during the entire disease course, as reported in other cohorts (56).

### Symptomatic MMD, MMS, and Incidental MMS: Is Asymptomatic Better?

Statistical comparison between symptomatic MMD (symtpMMD) and MMS (symptMMS) populations showed only one statistically significant difference about functional disability at follow-up (Table 2). Nevertheless, other parameters indicate a trend toward a more severe disease in patients with MMS (higher proportion of patients experiencing stroke after diagnosis and with intellectual disability at follow-up). This could also partially be due to their associated conditions.

Surprisingly, we registered an unexpectedly benign course in the small group of asymptomatic patients (incMMS). In spite of this, international data suggest that moyamoya is a progressive disorder even in initially asymptomatic cases, supporting the rationale of early surgical intervention to minimize morbidity from stroke, according to some authors (57). Larger cohorts are warranted to conclude about this topic.

## Limitations and Conclusions

Our study is severely limited by the retrospective and multicenter design, the relatively small cohort, the availability and completeness of information, and the reduced use of standardized measures (due to the study design, some subsets of data lacked of reproducible definitions).

The same multicenter nature of the study that prevented the collection of further and more accurate data, however, made the present study possible, an unprecedented effort in the field of pediatric moyamoya in Italy.

Our cohort showed that moyamoya typically has onset in preschool age in Italian children with a high proportion of cerebrovascular ischemic clinical events, but it may also manifest with multiple clinical phenotypes including headache (isolated or with other symptoms), seizures, movement disorder. We confirmed that the younger the age at disease onset, the more severe the disease presentation, course and outcome. A substantial proportion of children in our series had intellectual and motor problems at follow-up, despite a high rate of surgical corrections, confirming the severity of pediatric-onset moyamoya. On the other side, we observed a very benign course in a small group of children with incidentally diagnosed MMS, suggesting that moyamoya could be more diffuse than expected in children with a medical condition, especially sickle cell disease and other genetic diseases. In Italy, diagnostic and knowledge advances have improved timing of diagnosis and availability of neurosurgical management for pediatric moyamoya.

## Data Availability Statement

The raw data supporting the conclusions of this article will be made available by the authors, without undue reservation.

## Ethics Statement

Ethical review and approval was not required for the study on human participants in accordance with the local legislation and institutional requirements. Written informed consent for participation was not provided by the participants' legal guardians/next of kin because the study complied with the general ethical requirements for retrospective observational studies. In particular, no experimental interventions were performed and patient identity cannot be retrieved from the manuscript.

## Author Contributions

CP and SSar: collected data from the participating centers. RM: performed the neuroradiology review. AF and DA: performed the statistical analyses. CP and MN: prepared the manuscript. MZ, RP, GMi, DC, AR, ACo, IT, RC, PM, AI, PA, LGi, SSav, TF, GS, EL, FZT, GP, SC, FR, BS, SB, FG, DMC, LC, FTo, VB, FC, ASu, LS, MA, EC, LD, GD, GE, LGr, GMo, FN, FO, DP, PR, MR, ASp, PS, ASk, LL, ACa, and CM: provided patient's data. All authors have read and approved the final version of the manuscript.

## Conflict of Interest

The authors declare that the research was conducted in the absence of any commercial or financial relationships that could be construed as a potential conflict of interest.

## Publisher's Note

All claims expressed in this article are solely those of the authors and do not necessarily represent those of their affiliated organizations, or those of the publisher, the editors and the reviewers. Any product that may be evaluated in this article, or claim that may be made by its manufacturer, is not guaranteed or endorsed by the publisher.
