# Supplementary material for: Pediatric Moyamoya Disease and Syndrome in Italy: A Multicenter Cohort
Source: Front Pediatr. 2022 May 6;10:892445. doi: 10.3389/fped.2022.892445 (PMC9120837; doi:10.3389/fped.2022.892445)
Supplement: Supplementary file 1 [file Data_Sheet_1.docx]

**S1. Subset of children with symptom onset before 24 months of age**

| **#,**  **Sex** | **Race** | **MMD / MMS** | **Associated condition** | **Age at symptom presentation (months)** | **Age at diagnosis (months)** | **Disease type** | **AIS presentation with acute symptomatic seizures** | **Posterior circulation involvement at imaging** | **Neuro**  **SURGERY** | **Antiplatelet**  **THERAPY** | **AIS after diagnosis** | **TIA after diagnosis** | **Cognitive impairment at follow up** | **mRS >2 at follow-up** |
| --- | --- | --- | --- | --- | --- | --- | --- | --- | --- | --- | --- | --- | --- | --- |
| 1, F | Caucasian | MMD | - | 5 | 5 | AIS | Yes | No | Yes | No | No | No | No | 1 |
| 2, F | Caucasian | MMD | - | 11 | 11 | AIS | Yes | Yes | Yes | Yes | No | No | No | 3 |
| 3, F | Caucasian | MMD | - | 12 | 36 | TIAs | N.a. | N.a. | Yes | Yes | Yes | No | Yes | 1 |
| 4, M | Caucasian | MMD | - | 12 | 13 | AIS | No | Yes | Yes | Yes | Yes | No | No | 0 |
| 5, M | Caucasian | MMS | NF1 | 15 | 15 | AIS | No | Yes | Yes | Yes | Yes | Yes | Yes | 4 |
| 6, M | Caucasian | MMD | - | 17 | 29 | TIAs and seizures | N.a. | Yes | Yes | No | No | Yes | N.a. | Nd |
| 7, M | Caucasian | MMS | Syndrome with delay and neonatal seizures | 17 | 17 | AIS | Yes | Yes | Yes | Yes | Yes | No | Yes | Nd |
| 8, M | Caucasian | MMS | Renal artery stenosis | 17 | 17 | AIS | Yes | Yes | Yes | Yes | Yes | No | No | 1 |
| 9, M | Caucasian | MMD | - | 20 | 20 | AIS | Yes | Yes | Yes | Yes | No | No | Yes | 3 |
| 10, M | African | MMS | Suspected genetic syndrome | 21 | 22 | Seizures | N.a. | No | Yes | Yes | Yes | No | Yes | 2 |

S1. Legend: AIS: arterial ischemic stroke; MMD: moyamoya disease; MMS: moyamoya syndrome; mRS: modified Rankin Scale; N.a.: not available; NF1: Neurofibromatosis type 1; TIA: transient ischemic attack.

**S2. Complete neuroradiology report**

| **sex** | **ethnicity** | **associated condition** | **symptomatic vs incidental diagnosis** | **type of image (when different from MRI)** | **steno-occlusion**  **(s-o)** | | **networks** | | **s-o and networks R=right, L=left** | **posterior circulation involvement** | **ischemic lesions** | **hemorrhagic lesions** |
| --- | --- | --- | --- | --- | --- | --- | --- | --- | --- | --- | --- | --- |
|  |  |  |  |  | **right** | **left** | **right** | **left** |  |  |  |  |
| F | asian | yes | symptomatic |  | o | o | x | x | **RL RL** | x | **x** |  |
| M | caucasian | yes | symptomatic |  | o | o | x | x | **RL RL** | x | **x** |  |
| F | african | yes | symptomatic |  | o | o | x | x | **RL RL** | x | **x** |  |
| F | caucasian | yes | symptomatic |  | o |  | x |  | **R R** |  |  |  |
| M | caucasian | yes | symptomatic |  | o | o | x | x | **RL RL** |  |  |  |
| F | caucasian |  | symptomatic |  | o | o | x | x | **RL RL** |  | **x** |  |
| M | asian |  | symptomatic |  | o | o | x | x | **RL RL** |  | **x** |  |
| M | asian |  | symptomatic |  | o | o | x | x | **RL RL** | x | **x** |  |
| F | caucasian |  | symptomatic |  | o | o | x | x | **RL RL** | x | **x** |  |
| M | african | yes | symptomatic |  | o |  | x |  | **R R** |  | **x** |  |
| F | caucasian |  | symptomatic |  | o | o | x | x | **RL RL** | x |  |  |
| F | caucasian | yes | symptomatic |  | o | o | x | x | **RL RL** | x | **x** |  |
| M | caucasian |  | symptomatic |  | o | o | x | x | **RL RL** |  | **x** |  |
| M | caucasian | yes | symptomatic |  | o | o | x | x | **RL RL** | x | **x** |  |
| M | caucasian |  | symptomatic | angiography | o | o |  | x | **RL L** |  | **n.a.** | **n.a.** |
| F | caucasian | yes | symptomatic | angiography | o | o | x | x | **RL RL** |  | **n.a.** | **n.a.** |
| F | caucasian |  | symptomatic |  | o | o | x | x | **RL RL** |  | **x** |  |
| F | asian |  | symptomatic |  | o | o | x | x | **RL RL** | x | **x** |  |
| F | caucasian |  | symptomatic |  | o | o | x | x | **RL RL** | x | **x** |  |
| F | caucasian |  | symptomatic |  | o | o | x | x | **RL RL** | x | **x** |  |
| F | caucasian |  | symptomatic |  | o | o | x | x | **RL RL** | x | **x** |  |
| M | asian | yes | symptomatic | angiography | o | o | x | x | **RL RL** |  | **n.a.** | **n.a.** |
| F | caucasian |  | symptomatic |  | o | o | x | x | **RL RL** |  |  |  |
| M | caucasian | yes | symptomatic |  | o | o | x | x | **RL RL** | x | **x** |  |
| M | caucasian | yes | symptomatic |  | o | o | x | x | **RL RL** |  |  |  |
| M | caucasian |  | symptomatic |  | o | o | x | x | **RL RL** | x | **x** |  |
| F | caucasian | yes | symptomatic |  | o | o | x | x | **RL RL** | x | **x** |  |
| F | caucasian | yes | symptomatic |  | o | o | x | x | **RL RL** | x | **x** |  |
| M | caucasian |  | symptomatic |  | o | o | x | x | **RL RL** | x | **x** |  |
| F | african | yes | symptomatic |  | o | o | x | x | **RL RL** |  | **x** |  |
| M | caucasian |  | symptomatic |  | o | o | x | x | **RL RL** | x | **x** |  |
| M | caucasian | yes | symptomatic |  | o | o | x | x | **RL RL** | x | **x** |  |
| F | caucasian |  | symptomatic |  | o | o | x | x | **RL RL** | x |  |  |
| M | asian |  | symptomatic |  | o | o | x | x | **RL RL** | x | **x** |  |
| M | caucasian |  | symptomatic |  | o | o | x | x | **RL RL** | x |  |  |
| F | caucasian |  | symptomatic |  | o | o | x | x | **RL RL** | x |  |  |
| F | caucasian | yes | incidental |  | o |  | x |  | **R R** |  |  |  |
| F | caucasian | yes | incidental |  | o | o | x | x | **RL RL** |  | **x** |  |
| F | african | yes | incidental |  | o | o | x | x | **RL RL** |  |  |  |
| F | african | yes | incidental |  | o | o | x | x | **RL RL** |  |  |  |
| M | caucasian | yes | incidental |  |  | o |  | x | **L L** |  |  |  |
| F | caucasian | yes | incidental |  | o |  | x |  | **R R** |  |  |  |
| M | caucasian | yes | incidental |  | o |  | x |  | **R R** |  | **x** |  |
| M | asian | yes | incidental |  | o | o | x | x | **RL RL** |  |  |  |
| M | caucasian | yes | symptomatic | angiography | o |  | x |  | **R R** |  | **n.a.** | **n.a.** |
| F | caucasian | yes | incidental |  | o | o | x | x | **RL RL** | x |  |  |
| M | caucasian | yes | symptomatic |  | o | o | x | x | **RL RL** | x | **x** |  |
| M | caucasian | yes | symptomatic | angiography | o | o | x | x | **RL RL** |  | **n.a.** | **n.a.** |
| F | caucasian | yes | symptomatic |  | o | o |  | x | **RL L** |  | **x** |  |
| M | caucasian | yes | incidental |  |  | o |  | x | **L L** |  |  |  |
| F | asian | yes | incidental |  | o | o | x | x | **RL RL** |  | **x** |  |
| M | caucasian |  | symptomatic |  | o | o | x | x | **RL RL** | x | **x** |  |
| F | asian |  | symptomatic |  | o | o | x | x | **RL RL** | x | **x** |  |
| F | caucasian | yes | symptomatic |  | o | o | x | x | **RL RL** | x | **x** |  |
| F | caucasian |  | symptomatic |  | o | o | x | x | **RL RL** | x | **x** |  |
| F | caucasian | yes | incidental |  | o | o | x | x | **RL RL** |  | **x** |  |
| F | caucasian | yes | symptomatic |  | o | o | x | x | **RL RL** | x | **x** |  |
| M | caucasian |  | symptomatic |  | o | o |  | x | **RL L** |  | **x** |  |

Legend: F: female; L: left; M: male; n.a.: not available; R: right

**S3. Comparison of most recent European studies including children with moyamoya**

| **First author** | **Year of publication** | **Country** | **Study population** | **Number of children included** | **Aim of the study** | **Age at onset/diagnosis** | **Disease types** |
| --- | --- | --- | --- | --- | --- | --- | --- |
| Kraemer. | **2008** | Germany | Adults/Pediatrics MMD | 5 (age <20 yrs) | Clinical data of white population | Mean age at onset (total population): 31 years | Cerebral ischemic events (all patients) |
| Czabanka | **2011** | Germany | Adults/pediatrics MMD/MMS | 8 (age <20 yrs) | Angiographic effects after bilatrevasc. approach | Mean age at diagnosis: 14 years | TIA 6/8, TIAs 1/8, Stroke 1/8 |
| Kossorotoff | **2011** | France | Pediatric MMD/MMS | 53 | Epidemiological data in non-Asian population | - | - |
| Acker | **2014** | Germany | Adults/pediatrics MMD | 25 juveniles | Ethnicity variability in moyamoya presentation | Age at presentation: 9.2 years | Disease main types (among juveniles): ischemic events 72%, hemorrhagic events 12%, cognition 8%. |
| Santoro | **2016** | Italy-France | Pediatric MMS in NF1 | 18 | Clinical, radiologic, genetic characteristic of MMS in NF1 | Age at diagnosis: 7.4 years | Headache 6/18, Stroke 2/18, Seizures 2/18.  8/18 incidental diagnosis. |
| Lanterna | **2016** | Italy | Pediatric MMD, MMS unilat MMD | 34 children and adults (number of children not specified) | Application of bypass/combined surgical approach | Mean age at diagnosis (total population): 34 years | TIA or stroke (all children) |
| Blauwblomme | **2017** | Germany | Pediatric MMD and MMS | 64 | Long-term outcome and perioperative after MBH procedure | Age at surgery: 7 years.  Mean diagnostic delay: 4 months. | TIA 34%, Stroke 47%, Hemorrhage 6%, Other 12.5%. Asymtomatic: 12.5% |
| Tho-calvi | **2018** | United Kingdom | Pediatric MMD and MMS | 88 | Clinical features, course, and outcomes | Age at onset. 5.1 years.  Age at surgical treatment: 6.3 years | Stroke 41%, TIA 33%, Cerebral hemorrhage 1%, Seizure 5%, Headache 11%, Hemichorea 2%. Asymptomatic 7% |
| Mirone | **2019** | Italy | Pediatric MMD and MMS, unilateral MMD | 10 | Clinical and radiological outcome after multiple burr holes | Mean age at presentation: 8.5 years | Stroke 2/10, TIA 3/10, Seizures 1/10, Headache 1/10. Asymptomatic 3/10  (all population) |
| Bersano | **2019** | Italy | Pediatric and adult MMD and MMS, unilateral MMD | 25 | Genetic study (GENOMA project) | Mean age at presentation (all patients): 35.5 +/- 19.6 years | Stroke 65.3% (ischemic 76%, hemorrhagic 24%), TIA 12%, Headache 45%, Seizures 29%, Cognitive impairment 21%, Pychiatric disorders 20%, Ophthalmic disorder 10% |
| Savolainen | **2019** | Finland | Pediatric and adult MMD | Not available | Follow up |  |  |
| Birkeland | **2020** | Denmark | Pediatric and adult MMD | 52 (18/52 younger than 18 years at diagnosis) | Epidemiological population study | Bimodal distribution of age at presentation, with a peak in childhood and another in middle-aged adults. The average age at presentation was 7 years and 40 years in children and adults, respectively. | Ischaemic stroke (33%), Hemorrhage (23%), Headache (17%), TIA (14%)  (in the total adult and pediatric cohort) |
| Doherty | **2020** | Ireland | Pediatric and adult MMD and MMS | 8 (<18 years) | Demographics, clinical presentations and outcomes | Mean age at presentation (all patients): 19 years (2 months-49 years) | Ischemic stroke 4/8, TIAs 4/8 |

S3. Legend: MMD: moyamoya disease; MMS: moyamoya syndrome; NF1: neurofibromatosis type 1; TIA: transient ischemic attack

**S4. Comparison between the previous Italian study by Battistella et al. and our case series**

|  | **Battistella et al. 1997** | **Our study** |
| --- | --- | --- |
| **Number of patients** | 27 children (tot. 34) | 65 children |
| Unilateral cases | 10 children | - |
| **Clinical information** | | |
| **Age at onset** | 11.4 years (1-43 years) | 5.4 years (5m-16y) for *symptomatic patients* |
| **Age at diagnosis** | *total population* 13.5 years (2-55y) | 7.4 years (5m-24.5y) |
|  |  |  |
| **Mean diagnostic delay** | 25 months | 16.8 months |
| **Disease types** | *total population* |  |
| TIAs | 44% | 32% |
| Strokes | 15% | 43% |
| Seizures | 18% | 17% |
| Headache | 20% | 23% |
| Hemorrhagic infarction | 3% | 0 |
| Multiple phenotypes | Not considered | 32% |
| **Treatment** | | |
| **Medical treatment** |  | |
| Antiplatelet agents | 22% | 69% |
| Calcium channel blockers | 16% | 5% |
| Vasodilators | 14% | 9% |
| **Surgical treatment** | 5/27 = 18.5% | 73% |
| **Outcomes** | | |
| Follow up duration | Mean 6 years (1-15 years) | Mean 5.1 years (0.5-15 years) |
| **Motor impairment** | 47% | 43% |
| **Intellectual deterioration** | 41% | 31% |
| **Death** | 3% | 0% |
